# *nor* 3′-Demethoxyisoguaiacin from *Larrea tridentata* Is a Potential Alternative against Multidrug-Resistant Bacteria Associated with Bovine Mastitis

**DOI:** 10.3390/molecules27113620

**Published:** 2022-06-05

**Authors:** Ana Lizet Morales-Ubaldo, Manases Gonzalez-Cortazar, Adrian Zaragoza-Bastida, Martín A. Meza-Nieto, Benjamín Valladares-Carranza, Abdulrahman A. Alsayegh, Gaber El-Saber Batiha, Nallely Rivero-Perez

**Affiliations:** 1 Área Académica de Medicina Veterinaria y Zootecnia, Instituto de Ciencias Agropecuarias, Universidad Autónoma del Estado de Hidalgo, Rancho Universitario Av. Universidad Km 1, EX-Hda de Aquetzalpa, Tulancingo 43600, Hidalgo, Mexico; mo260116@uaeh.edu.mx (A.L.M.-U.); martin_meza3292@uaeh.edu.mx (M.A.M.-N.); 2 Centro de Investigación Biomédica del Sur, Instituto Mexicano del Seguro Social, Argentina No. 1. Col. Centro, Xochitepec 62790, Morelos, Mexico; gmanases2000@gmail.com; 3 Centro de Investigación y Estudios Avanzados en Salud Animal, Facultad de Medicina Veterinaria y Zootecnia, Universidad Autónoma del Estado de México, Km 15.5 Carretera Panamericana Toluca-Atlacomulco, Toluca 50200, Estado de Mexico, Mexico; bvalladaresc@uaemex.mx; 4 Clinical Nutrition Department, Applied Medical Sciences College, Jazan University, Jazan 82817, Saudi Arabia; aalsayegh@jazanu.edu.sa; 5 Department of Pharmacology and Therapeutics, Faculty of Veterinary Medicine, Damanhour University, Damanhour 22511, Egypt; gaberbatiha@gmail.com

**Keywords:** *Larrea tridentata*, *nor*-3 demethoxyisoguaiacin, antibacterial activity, multidrug-resistance, bovine mastitis

## Abstract

Bovine mastitis is one of the most common diseases in dairy cows, and it causes significant economic losses in dairy industries worldwide. Gram-positive and Gram-negative bacteria can cause bovine mastitis, and many of them have developed antimicrobial resistance. There is an urgent need for novel therapeutic options to treat the disease. *Larrea tridentata*-derived compounds represent an important potential alternative treatment. The aim of the present study was to isolate and characterize antibacterial compounds from *Larrea tridentata* against multidrug-resistant bacteria associated with bovine mastitis. The *L. tridentata* hydroalcoholic extract (LTHE) exhibited antibacterial activity. The extract was subjected to a bipartition, giving an aqueous fraction (moderate antibacterial activity) and an organic fraction (higher antibacterial activity). Chromatographic separation of the organic fraction enabled us to obtain four active sub-fractions. Chemical analyses through HPLC techniques were conducted for the LTHE, fractions, and sub-fraction Ltc1-F3, from which we isolated two compounds, characterized by ^1^H and ^13^C NMR analyses. Compound *nor*-3 demethoxyisoguaiacin exhibited the best antibacterial activity against the evaluated bacteria (MIC: 0.01–3.12 mg/mL; MBC: 0.02–3.12 mg/mL). The results indicated that *nor*-3 demethoxyisoguaiacin can be used as an alternative treatment for multidrug-resistant bacteria associated with mastitis.

## 1. Introduction

Bovine mastitis is defined as an inflammation of the udder, and it is the most common and challenging disease in dairy animals, causing economic losses due to the costs of treatment, herdsman time, decreased milk production, decreased milk quality, increased culling, loss of premiums, pre-term drying-off, and animal welfare [1,2,3,4]. An infectious etiology is usually the most prevalent; although fungi, viruses, and algae can cause mastitis, bacteria are the most common pathogens in the mammary glands of cows [1,5].

Both Gram-positive and Gram-negative bacteria can cause mastitis and have been categorized as contagious, environmental, and opportunistic pathogens [1,2,3]. *Staphylococcus aureus*, the main contagious bacterium, lives on the udder and teat skin, colonizing and growing into the teat canal, and is transmitted from infected to uninfected teats during the milking process [1,6,7]. Among the environmental pathogens are *Escherichia coli* and *Klebsiella pneumoniae*, which live in the bedding and housing of the herd and tend to enter the teat canal during the milking process [1,6,7].

Other less common mastitis-causing organisms are *Listeria monocytogenes*, *Bacillus cereus*, *Pseudomonas aeruginosa*, and *Pasteurella multocida*. They are better described as opportunistic bacteria, looking for a chance to colonize the lining of the teat or udder skin [1,6,7,8,9,10].

The complex etiology and multi-factorial nature of this disease make it difficult to control. Antimicrobials such as penicillin, cephalosporin, streptomycin, and tetracycline are often administered in the dairy industry to treat and prevent mastitis [1,11,12].

Other studies have reported that antimicrobials such as amoxicillin, hetacillin, pirlimycin, ceftiofur, and cephapirin can be used intramammarily to treat cows for clinical mastitis, although the most common systemic antimicrobials are ampicillin, ceftiofur, oxytetracycline, penicillin, or a combination of spectinomycin and lincomycin [13].

According to data collected by Tomazi and Dos Santos in 2020, antimicrobials such as cephoperazone, cefquinome sulfate, ceftiofur hydrochloride, ciprofloxacin, gentamicin, oxytetracycline, neomycin, spiramycin neomycin, sulfadiazine, and trimethoprim are used intramammarily, and among the available systemic treatments are amoxicillin trihydrate, cefquinome sulfate, ceftiofur hydrochloride, enrofloxacin, florfenicol, gentamicin, gentamicin/amoxicillin, norfloxacin, oxytetracycline, sulfadiazine/trimethoprim, and tylosin [14].

The literature shows that several active ingredients exist to treat cows for mastitis; however, their misuse can drive antimicrobial resistance. Recent studies have reported that bacteria associated with mastitis show resistance to antimicrobials such as oxacillin, enrofloxacin, tetracycline, trimethoprim–sulfamethoxazole, gentamicin, erythromycin, lincomycin, amoxicillin, ceftiofur, penicillin, marbofloxacin, and cefoxitin [10,12].

Antimicrobial resistance occurrence must be monitored to reduce this phenomenon in the dairy cattle industry. Some authors have reported on the urgent need for alternative antimicrobial therapies to treat and prevent the disease, given the widespread emergence of antibiotic resistance. Naturally derived compounds represent an important option for the development of effective antimicrobials against resistant bacteria [12,15,16,17,18,19].

Several studies have demonstrated that plant extracts are able to act against bacteria such as *S. aureus*. Gomes et al. (2021) reported that *Eucalyptus globulus* shows bacteriostatic activity against said bacterium. In a similar study, the antibacterial activity of *Hypericum perforatum* against mastitis pathogens was determined. The potential use of copalic acid to control Gram-positive bacteria associated with mastitis has also been reported [20,21,22].

Therefore, *Larrea tridentata* and its compounds represent potential alternative treatments for bovine mastitis. *L. tridentata*, a perennial shrub, is a botanical species well known in Mexico, mainly for its applications in traditional medicine [23,24]. Recent studies have demonstrated its pharmacological properties [25,26,27,28], including antibacterial activity [29,30,31].

Previous studies have reported that extracts from *L. tridentata* show antibacterial activity against a wide range of sensitive and antimicrobial-resistant bacterial strains, in the same way that pure compounds such as nordihydroguaiaretic acid, *meso* dihydroguaiaretic acid, dihydroguaiaretic acid 4-epi-larreatricin, among others, exhibit antibacterial effects, which sometimes could enhance and potentialize some other antibiotics [32,33,34,35,36,37,38,39,40,41]. The aim of the present study was to isolate and characterize antibacterial compounds from *Larrea tridentata* against multidrug-resistant bacteria associated with bovine mastitis.

## 2. Results and Discussion

### 2.1. Antimicrobial Sensitivity Test of Multidrug-Resistant Clinical Isolates

According to the results of an antimicrobial sensitivity test, the bacteria evaluated in the present study were classified as multidrug-resistant, because all of them showed resistance to at least three non-related antimicrobials (Table 1) [42,43].

### 2.2. Antibacterial Activity

As can be seen in Table 2, LTHE inhibited the growth of all strains under study, in a range of concentrations from 0.19 to 25.0 mg/mL.

Among the reference strains, the most sensitive bacterium was *S. aureus*^6538^ (0.39 mg/mL) (*p* = 0.0001), followed by *L. monocytogenes*^19113^ (3.12 mg/mL), whereas *E. coli*^35218^ and *P. aeruginosa*^9027^ were the least sensitive strains, obtaining MIC values of 12.5 mg/mL for both strains.

Regarding MDR clinical isolates, LTHE showed better activity against Gram-positive strains (*S. aureus* and *B. cereus*), with MIC values from 0.19 to 0.78 mg/mL. Among Gram-negative bacteria, the most sensitive strain was *P. multocida* (0.78 mg/mL), whereas against *E. coli* strains and *K. pneumoniae,* concentrations from 6.25 to 25 mg/mL were determined (Table 2) (*p* = 0.0001).

A study carried out by Gerstel et al. (2018) tested the antibacterial activity of *L. tridentata* hydroalcoholic extract (75:25, ethanol:water), against nonantibiotic-resistant and antibiotic-resistant *S. aureus* strains, finding MIC values from 0.35 to 15 µg/mL. Even though these results cannot be compared with ours due to differences in methodology, the activity of *L. tridentata* hydroalcoholic extracts against these kinds of bacteria support our results [32].

Bocanegra-García et al. (2009) determined that four different extracts from aerial parts of *L. tridentata* showed activity at concentrations higher than 0.250 mg/mL against *S. aureus*. Similar concentrations were determined for *S. aureus* strains in this study (0.19–0.39 mg/mL). Martins et al. (2013) obtained a concentration of 0.12 mg/mL against said bacterium, similar to that obtained against *S. aureus*^02^ (0.19 mg/mL) [34,45].

An MIC range from 0.125 to >0.250 mg/mL was determined against *L. monocytogenes*, lower concentrations than determined in the present study (3.12 mg/mL) [45].

Bocanegra-*García* et al. (2009) found no antibacterial activity against *B. cereus*, *E. coli*, *P. aeruginosa,* and *K. pneumoniae*, unlike in our study; we found that LTHE showed inhibitory activity against said bacteria (MIC values = 0.78 to 25 mg/mL) [45].

Prior studies have demonstrated that *L. tridentata* extracts can inhibit the growth of *S. aureus*, *B. subtilis*, *S. gallinarum*, *Salmonella enterica E. coli*, *P. aeruginosa*, *Klebsiella oxytoca,* and *K. pneumoniae*. Despite the use of different methodologies, the antibacterial activity of *L. tridentata* can be confirmed [29,34,46].

#### 2.2.1. Chemical Characterization of the Extract

In the present study, qualitative characterization tests were carried out on the hydroalcoholic extract of *L. tridentata*, indicating the presence of unsaturations, phenolic oxydryls (vegetable tannins), coumarins, lactones, flavonols, sesquiterpenlactones, and steroids. Moreover, LTHE was positive to bicarbonate and floratanino tests.

The analysis in the gas chromatograph of *L. tridentata* hydroalcoholic extract determined the presence of thymol and carvacrol in concentrations of 5.3840 mg/mL and 4.1840 mg/mL, respectively, without determining its presence of terpinene, limonene, and linalool.

Most of the abovementioned compounds were reported as constituents of *L. tridentata*, which are associated with bacteriostatic or bactericidal activity against important pathogens, proceeding through different modes of action [29,30,35,47,48,49,50,51,52].

Part tannins can induce complexation with microbial enzymes or substrates, generating membrane damage, amino acid limitations, energy metabolism disorder, and iron deprivation [53,54].

Some studies have suggested that modes of action of flavonoids are related to cell lysis and increases in membrane permeability, inhibition of nucleic acid synthesis, interference with energy metabolism and biofilm formation, inhibition of porins, and reduction in pathogenicity [55,56,57,58].

With respect to thymol and carvacrol, the mechanism of action is related to the inhibition of biofilm formation and cell membrane damage [59,60,61]. Moreover, a recent study reported that said compounds can cause disruption of the membrane, inhibition of efflux pumps, interference in the formation of biofilms, inhibition of bacterial motility, and the inhibition of membrane ATPases [62].

#### 2.2.2. Identification of Major Compounds

**Figure 1:**
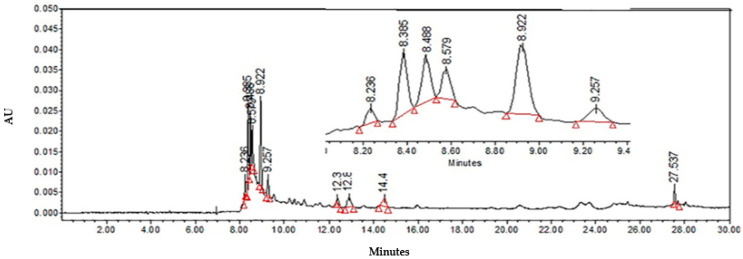
HPLC chromatogram at 350 nm of *Larrea tridentata* hydroalcoholic extract.

In order to identify the compounds in LTHE, HPLC analysis was performed, showing the presence of seven peaks, which corresponded to: coumarin (8.236 min), caffeic acid derivatives (8.385, 12.400, and 12.900 min), flavonols: rutin (8.922 min), quercetin glucoside (9.257 min), and kaempferol (14.483), and a peak with a retention time of 27.537 min corresponding to the compound *nor* 3′-demethoxyisoguaiacin (Figure 1).

Antibacterial activities of these compounds were previously reported. De Souza et al. (2005) reported that coumarin and 45 coumarin derivatives are active against Gram-positive and Gram-negative bacteria. Similarly, De la Cruz-Sánchez et al. (2019) reported that *Morinda citrifolia* antibacterial activity was associated with coumarin, identified as scopoletin [63,64].

With respect to the mechanism of action, one study suggested that some coumarins interact with bacterial DNA, causing an important decrease in topoisomerase activity, affecting the replication and transcription of DNA, interfering with the synthesis of proteins, and inhibiting bacterial replication. These mechanisms of action may be similar for coumarins present in LTHE [65].

Regarding caffeic acid derivatives, several studies have reported their antibacterial activity. Phytochemicals are potent bactericidal compounds that can eliminate bacterial growth through an oxidative stress mechanism [66,67,68,69].

In the same sense, some authors have found that rutin shows antibacterial activity against both Gram-positive and Gram-negative bacteria [70,71,72,73]. Possible mechanisms of action could be related to an increase in the permeability of the bacterial cell wall and cytoplasmic membrane. Anath et al. (2015) explained that rutin has the capacity to penetrate the outer membrane through porin and liberate hydroxyl radicals, causing oxidative stress, which provokes bacterial death [74,75].

Similarly, quercetin glucoside has been considered as an antibacterial compound, effective against MDR Gram-negative strains [76].

The presence of phenolic compounds, such as NDGA, kaempferol, and quercetin, was previously reported in *L. tridentata* and associated with its antibacterial activity. Some authors have stated that, due to lipophilic characteristics, phenolic compounds accumulate in the lipid bilayer of the membrane, increasing permeability [34,75].

Evaluation of LTHE fractions showed that the aqueous fraction (LTAq-F) possessed inhibitory activity against all reference strains at a concentration range from 1.56 to 3.12 mg/mL. On the other hand, against MDR clinical isolates, this fraction only showed an inhibitory effect against *E. coli* (12.5–25.0 mg/mL) and *K. pneumoniae* (6.25 mg/mL) (Table 2).

The obtained data showed that bacteria were more sensitive to the organic or ethyl acetate fraction (LTEtOAc-F); with respect to the reference strains, this treatment showed better activity against *S. aureus*^6538^ and *L. monocytogenes*^19113^ (0.04 mg/mL), whereas against *E. coli*^35218^ and *P. aeruginosa*^9027^, a concentration of 3.12 mg/mL was determined.

Regarding Gram-positive MDR clinical isolates, a range of concentrations from 0.19 to 0.39 mg/mL was determined. Among Gram-negative strains, *P. multocida* was the most sensitive bacterium (MIC = 0.39 mg/mL), whereas for the rest of the bacteria, a concentration of 3.12 mg/mL was determined (Table 2).

In a recent study, the *L. tridentata* ethyl acetate fraction showed better activity against Gram-positive and Gram-negative bacteria than hydroalcoholic extract, which was similar to the results obtained in the present study [31].

Martins et al. (2013) determined that the ethyl acetate fraction of *L. tridentata* inhibited the growth of *S. aureus*, *E. coli*, *P. aeruginosa*, and *K. pneumoniae*. Although different techniques were used in both studies, our results were supported [34].

Martins et al. determined MIC values from 0.0625 to 0.125 mg/mL for *S. aureus* strains when the ethyl acetate fraction was evaluated, which are very close to those determined in the present study against *S. aureus*^6538^ (0.04 mg/mL) and *S. aureus*^02^ (0.19 mg/mL) [34].

Ethyl acetate polarity allows us to perform better extractions, obtaining higher concentrations of bioactive compounds, which can easily pass through bacterial walls, explaining why better activity was observed with the *L. tridentata* organic fraction [77,78,79].

**Figure 2:**
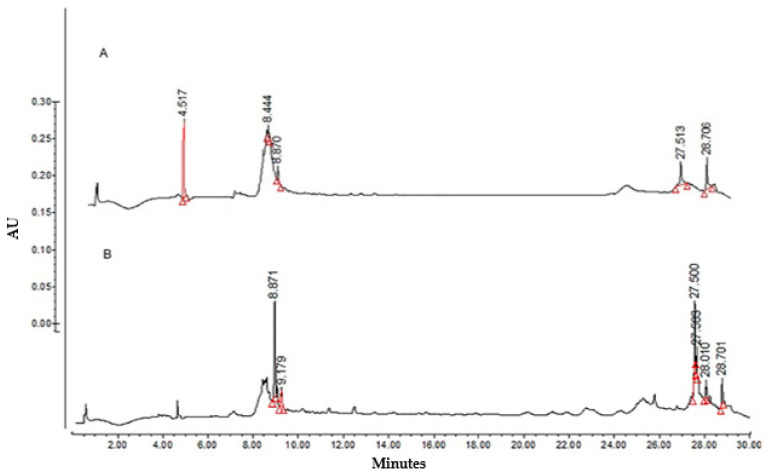
HPLC chromatograms of (**A**) *L. tridentata* aqueous fraction and (**B**) *L. tridentata* organic fraction (280 nm).

HPLC analyses were performed to determine the compounds contained in both fractions. Compared with LTHE (Figure 1), the aqueous fraction (Figure 2A) did not show peaks corresponding to flavonols and caffeic acids derivatives. Nevertheless, *nor*-3 demethoxyisoguaiacin (27.513 min) was observed, although in lower concentration than in the organic fraction (Figure 2B) (27.500 min), in which caffeic acid derivatives (8.871 min) and quercetin–glucoside (9.179 min) were also present.

Based on the above, the inhibitory activity determined for the aqueous fraction was associated with the presence of *nor* 3′-demethoxyisoguaiacin.

Stronger activity was determined for the organic fraction, which could be associated with the synergy of the compounds identified in this fraction (*nor* 3′-demethoxyisoguaiacin, caffeic acid derivatives, and quercetin glucoside). Some authors have stated that secondary metabolites can act antagonistically or synergistically [80,81,82].

The organic fraction showed better activity than the aqueous; therefore, it was fractionated, obtaining seven sub-fractions, from which only four were evaluated (Ltc1 F3-F6).

The results showed that all four sub-fractions were active at a concentration of 0.09 mg/mL against *S. aureus*^6538^ and *L. monocytogenes*^19113^, except for Ltc1-F5, which was the best treatment for *S. aureus*^6538^, with an MIC value of 0.04 mg/mL (Table 2).

Against *E. coli*^35218^, Ltc1-F3 exhibited the highest activity, at a concentration of 1.56 mg/mL, whereas the rest of the sub-fractions were active at a concentration of 3.12 mg/mL, the same concentration as determined for *P. aeruginosa*^9027^ (Table 2).

With respect to Gram-positive MDR clinical isolates, *S. aureus*^01^ was sensitive to three sub-fractions at a concentration of 0.02 mg/mL. The least active treatment for this strain was Ltc1-F6 (0.78 mg/mL). Different concentrations were determined for *S. aureus*^02^; the best treatment was Ltc1-F3 (0.02 mg/mL), and the worst was Ltc1-F6 (0.39 mg/mL). The same concentrations were determined for *B. cereus* (Table 2).

With respect to *E. coli*^01^ and *E. coli*^02^, a concentration of 0.78 mg/mL was determined for Ltc1F3, F4, and F5, but concentrations of 3.12 and 6.25 mg/mL, respectively, were determined for Ltc1-F6. Lower inhibitory concentrations were determined for *K. pneumoniae*; an MIC value of 0.39 mg/mL was determined for the three first sub-fractions, and similar to *E. coli* strains, this bacterium was least sensitive to Ltc1-F6 (3.12 mg/mL).

The most sensitive Gram-negative MDR clinical isolate was *P. multocida*; the most active treatments sub-fractions were Ltc1-F3 and F5 (0.09 mg/mL), followed by Ltc1-F4 (0.19 mg/mL) and Ltc1-F6 (0.78 mg/mL) (Table 2).

The evaluated bacteria were most sensitive to Ltc1-F3; therefore, chemical characterization of was performed, allowing the identification of compound **1** (*nor* 3′-demethoxyisoguaiacin, from Ltc3-F9) and compound **2** (NDGA mixture, from Ltc3-F15).

Regarding the reference strains, the most sensitive bacterium to compound 1 was *L. monocytogenes*^19113^ (0.09 mg/mL), followed by *E. coli^35218^* (1.56 mg/mL) and *P. aeruginosa*^9027^ (3.12 mg/mL) (Table 2).

With respect to the other group, the most sensitive strains were *S. aureus*^02^ and *B. cereus* (0.01 mg/mL), followed by *S. aureus*^01^ (0.02 mg/mL). With respect whereas Gram-negative bacteria, a concentration of 0.78 mg/mL was determined for *E. coli*^01^, while against *E. coli*^02^ and *K. pneumoniae*, an MIC value of 0.39 mg/mL was determined. The lowest MIC was determined against *P. multocida* (0.09 mg/mL).

Regarding C**2**, the NDGA mixture only showed inhibitory activity against *P. aeruginosa*^9027^, but in higher concentrations than C**1**, because an MIC value of 1.56 mg/mL was determined.

**Figure 3:**
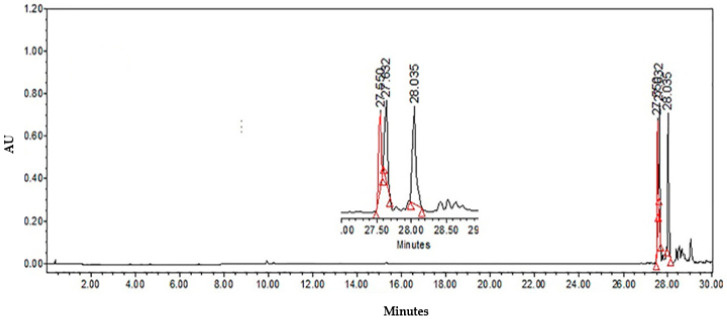
HPLC chromatogram at 280 nm of sub-fraction Ltc1-F3.

HPLC analysis of Ltc1-F3 was carried out, identifying three peaks with retention times of 27.550, 27.632, and 28.035 min, corresponding to the major compound *nor* 3′-demethoxyisoguaiacin (Figure 3).

Analysis of spectroscopic data (Table 3) of ^1^H and ^13^C NMR spectra and comparisons with previous results [28] allowed us to determine that this compound corresponded to *nor* 3′-demethoxyisoguaiacin.

Lignan was isolated for the first time from leaves and small twigs of the plant. The authors found that it showed fungicidal activity. According to Konno et al. (1990), this compound is also known as 3′-demethoxy-6-*0*-demethylisoguaiacin [83,84].

Recently, Bashyal et al. (2017) isolated this same compound from *L. tridentata*, and the authors found that *nor* 3′-demethoxyisoguaiacin showed anti-parasitic activity against *Giardia lamblia* and *Entamoeba histolytica* [28].

Regarding the antibacterial activity of *nor* 3′-demethoxyisoguaiacin, a previous study found antibacterial activity against 12 different bacteria, including *S*. *aureus*, *L*. *monocytogenes*, *E*. *coli*, *K*. *pneumoniae*, and *P*. *aeruginosa* [36].

Antibacterial activity was determined against *S. aureus* strains at concentrations from 0.0125 to 0.025 mg/mL, close to those determined for *S. aureus*^01^ and *S. aureus*^02^ (MIC values = 0.01 to 0.02 mg/mL). On the other hand, higher concentrations were determined for *E. coli* strains in our study (0.39–1.56 mg/mL), as compared with 0.05 mg/mL [36].

The mechanism of action of the isolated compound in the present study was previously determined; this compound showed activity against the cell membrane, repressing proteins of the ATP-binding cassette transport system, which causes bacterial death. The same compound was isolated in both studies; therefore, we suggest that the antibacterial activity reported in the present study is associated with the same mechanism of action [37].

With respect to compound **2**, NDGA has been reported as an important bioactive compound from *L. tridentata*, and is associated with antibacterial activity [34,48].

In the study carried out by Guzmán-Beltrán et al. (2016), NDGA in high concentrations inhibited mycobacterial growth. The results of another study showed that this compound, in combination with commercial antibiotics (aminoglycosides), showed enhanced antibacterial activity against both drug-sensitive and drug-resistant Gram-positive bacteria. The combination damaged the bacterial cell membrane, and a similar mechanism of action may have been exhibited in the present study against *P. aeruginosa*^9027^ [39,41].

In the present study, the minimal bactericidal concentrations were also determined. The results showed that LTHE had bactericidal activity against all evaluated bacteria at concentrations from 0.39 to 50.0 mg/mL.

With respect to the reference strains, the most sensitive was *S. aureus*^6538^ (0.78 mg/Ml), followed by *L. monocytogenes*^19113^ (6.25 mg/mL). With respect to Gram-negative strains, a concentration of 25 mg/mL was determined for both strains (Table 4).

The most sensitive MDR clinical isolates were *S. aureus*^01^*, S. aureus*^02^, and *B. cereus* (0.39–1.56 mg/mL). With respect to *E. coli* strains and *K. pneumoniae,* the MBC ranged from 12.50 to 50 mg/mL. A lower concentration was determined against *P. multocida* (1.56 mg/mL) (Table 4).

The bactericidal activity of *L. tridentata* extracts has previously been reported, but only against Gram-positive bacteria (*S. aureus*). It was not reported at which concentration this activity was determined. In the present study, bactericidal activity was determined against both Gram-positive and Gram-negative bacteria, and the concentrations were obtained [32,85].

In a recent study, the bactericidal activity of a hydroalcoholic extract of *L. tridentata* against Gram-positive and Gram-negative bacteria was determined at concentrations from 0.78 to 12.5 mg/mL [31].

With respect to fractions, the aqueous fraction only showed activity against the two Gram-positive reference strains at a concentration of 3.12 mg/mL, whereas the organic fraction exhibited bactericidal effects against all evaluated bacteria.

*S. aureus*^6538^ and *L. monocytogenes*^19113^ were sensitive at 0.09 mg/mL, and a higher concentration was determined for *E. coli*^35218^ and *P. aeruginosa*^9027^ (6.25 mg/mL).

Concentrations from 0.39 to 0.78 mg/mL were determined for Gram-positive MDR clinical isolates, whereas a concentration of 6.25 mg/mL was determined for all Gram-negative strains, except for *P. multocida*, for which an MBC of 0.78 mg/mL was determined.

A recent study supported our results; the authors determined that an *L. tridentata* organic fraction exhibited better bactericidal effects than an aqueous fraction [31].

With respect to the bactericidal activity of *L. tridentata* sub-fractions, it was observed that *S. aureus*^6538^ was sensitive to Ltc1-F5 (0.19 mg/mL) and Ltc1-F6 (0.09 mg/mL), but not to Ltc1-F3 and F4. Similar effects were observed for *L. monocytogenes*^19113^, because this bacterium also was sensitive to Ltc1-F3 and Ltc1-F6 at a concentration of 0.09 mg/mL. No bactericidal activity was determined for the other two sub-fractions (Table 4).

*E. coli*^35218^ was sensitive to three of the evaluated sub-fractions, with MBC values from 3.12 to 6.25 mg/mL. No bactericidal activity was determined for Ltc1-F3. Several sub-fractions showed important bactericidal activity against *P. aeruginosa*^9027^; treatments with Ltc1-F4, F5, and F6 were active at 3.12 mg/mL, and Ltc1-F3 at a concentration of 6.25 mg/mL (Table 4).

Regarding MDR clinical isolates, both *S. aureus* strains were sensitive to Ltc1-F3 and Ltc1-F4 at a concentration of 0.09 mg/mL. Some differences between strains were observed when Ltc1-F5 was evaluated; MBC values of 0.09 and 0.19 mg/mL were determined for *S. aureus*^01^ and *S. aureus*^02^, respectively, and only *S. aureus*^01^ was sensitive to Ltc1-F6 (1.56 mg/mL).

Against *B. cereus,* the most active treatments were Ltc1-F3 and Ltc1-F4 (0.04 mg/mL), followed by Ltc1-F5 (0.09 mg/mL). This bacterium was least sensitive to Ltc1-F6 (0.78 mg/mL) (Table 4).

For both *E. coli* strains and *K. pneumoniae*, Ltc1-F3 and Ltc1-F4 were active at 3.12 mg/mL, and a concentration of 6.25 mg/mL was determined for Ltc1-F5 and Ltc1-F6. Against *P. multocida*, concentrations of 0.19, 0.39, 0.78, and 1.56 mg/mL were determined for Ltc1-F3, F4, F5, and F6, respectively (Table 4).

Regarding isolated compounds, *S. aureus*^6538^ was not sensitive to any of them, *L. mononocytigenes*^19113^ (0.09 mg/mL) and *E. coli*^35218^ (1.56 mg/mL) were only sensitive to *nor* 3′-demethoxyisoguaiacin, and *P. aeruginosa* was sensitive to both compounds (3.12 mg/mL).

The MDR clinical isolates were only sensitive to *nor* 3′-demethoxyisoguaiacin; *S. aureus*^01^ and *S. aureus*^02^ were sensitive at concentrations of 0.04 and 0.02 mg/mL, respectively. The compound was also active against *B. cereus* at a concentration of 0.78 mg/mL. With respect to Gram-negative strains, an MBC value of 1.56 mg/mL was determined against both *E. coli* strains and *K. pneumoniae*, whereas *P. multocida* was sensitive at 0.19 mg/mL.

To date, few studies have determined the MBC values of plant extracts, fractions, or their compounds, and specifically of *L. tridentata*, which limits the discussion of our results. Nevertheless, some bio-guided studies, such as those performed by González-Alamilla et al. (2019) and Olmedo-Juárez et al. (2019), in which *Salix babylonica* and *Caesalpinia coriaria* sub-fractions and pure compounds showed bactericidal activity, support our results [80,86].

The results of calculations of the MBC/MIC ratio showed that LTHE and the organic fraction had bactericidal activity against all evaluated bacteria, whereas the aqueous fraction exhibited bactericidal effects against *S. aureus*^6538^ and *L. monocytogenes*^19113^.

For sub-fraction Ltc1-F3, bactericidal effects were only determined for *L. monocytogenes*^19113^ and *P. aeruginosa*^9027^. Among the MDR clinical isolates, bacteriostatic activity was determined for all strains, except for *B. cereus*, *E. coli*, and *P. multocida.*

Ltc1-F4 and F5 exhibited bactericidal effects against *E. coli*^35218^ and *P. aeruginosa*^9027^. With respect to MDR clinical isolates, Ltc1-F4 showed bactericidal effects against *S. aureus*^02^, *B. cereus*, *E. coli*, and *P. multocida*, whereas Ltc1-F5 was bactericidal against *S. aureus*^02^ and *B. cereus.* Ltc1-F6 showed bactericidal effects against all evaluated strains, except for *S. aureus*^02^.

*nor* 3′-demethoxyisoguaiacin was bactericidal against all evaluated bacteria, except *B. cereus.* Finally, the NDGA mixture only exhibited a bactericidal effect against *P. aeruginosa^9027^*.

In general, the evaluated treatments were considered bactericidal against most of the bacterial strains evaluated in the present study. Previously, some authors had indicated that such bactericidal compounds are desired and preferred. *nor* 3′-demethoxyisoguaiacin represents an important alternative treatment for both antibiotic-sensitive and multidrug-resistant bacteria. Moreover, some of the evaluated bacteria against which it showed effectiveness are in the critical priority group of antibiotic-resistant pathogens [87,88,89].

## 3. Materials and Methods

### 3.1. Plant Material

The aerial parts of *Larrea tridentata* were collected from wild-growing shrubs (different phenological stages) in the municipality of Matehuala, San Luis Potosí, Mexico (23°38′47″ N 100°30′40″ O), during the months of May, June, and July of 2019.

Temperatures during harvest months were as follows: May, maximum, 36 °C, minimum, 9 °C, mean, 23.1 °C; June, maximum, 33.7 °C, minimum, 9.9 °C, mean, 21.6 °C; and July, maximum, 31.7 °C, minimum, 11 °C, mean, 20.3 °C.

The plants were verified as *Larrea tridentata* (IBUNAM: MEXU: 1249920). Aerial parts of *L. tridentata* were dried in the dark at room temperature.

### 3.2. Preparation of Hydroalcoholic Extract

Dried aerial parts from *L. tridentata* (1500 g) were subjected to an extraction process through the maceration technique using 6 L of a hydroalcoholic solution of water/ethanol (60:40 *v*/*v*) at room temperature for 24 h. Subsequently, it was filtered using Whatman filter paper (Whatman^®^ 42), the solvent was eliminated using a rotary evaporator to obtain a semisolid extract (Büchi R-300, Flawil, Switzerland), and the extract was lyophilized before being stored at 4 °C until antibacterial evaluation, according to the methodology described by Rivero-Perez et al. (2019) [90]. A total amount of 138.64 g of LTHE was obtained.

### 3.3. Chemical Characterization of the Extract

A qualitative chemical profile was performed for the *L. tridentata* hydroalcoholic extract, according to the method of Rivero-Perez et al. (2019) [90].

The tests were as follows: test for unsaturations (KMnO_4_), test for phenolic oxydryls (FeCl_3,_ vegetable tannins), test for sterols and triterpenes (Liebermann–Burchard), Salkowski test for sterols and triterpenes, coumarins test, Baljet test for sesquiterpenlactones, H_2_SO_4_ test for flavonoids, Shinoda test for flavonoids, Dragendorff test for alkaloids, tannin test, floratanino test, steroid test, agitation test, sodium bicarbonate test, and Salkowski test for saponins.

The chemical composition of the hydroalcoholic extract of *L. tridentata* was determined according to the methodology described by Rivero-Perez et al. (2019), using a gas chromatograph (CG; Agilent Technologies series 6890 N, manufactured in the USA), with a polar column DB_WAXetr, at 250 °C and 12.13 psi, with a 36.5 mL min^−1^ flow of He after injection.

Conditions for the column were as follows: initial temperature 50 °C, from 0 to 2 min, increase of 10 in 10 °C up to 250 °C, constant for 5 min, reduction to 50 °C for 2 min with a flow of 1.6 mL of He min^−1^ at 12.13 psi, and average velocity of 25 cm s^−1^. A flame ionization detector (FID) was used at 210 °C with a 40 mL min^−1^ flow of H_2_ and a 450 mL min^−1^ flow of air. Standards (Sigma-Aldrich, St. Louis, MO, USA) used included thymol, carvacrol, linalool, terpinene, and limonene at different concentrations [90].

### 3.4. Identification of Major Compounds

The hydroalcoholic extract was chemically fractionated with the following methodology, published by González-Alamilla et al. (2019) and Olmedo-Juárez et al. (2019) [80,86].

*L. tridentata* hydroalcoholic extract (LTHE) was processed for bipartition via liquid–liquid chromatography, using water/ethyl acetate (1:3) solvent (Merck, Darmstadt, Germany), obtaining an aqueous (LTAq-F) and an organic fraction (LTEtOAc-F). Solvents were eliminated by low-pressure distillation (Büchi R-300, Flawil, Switzerland). Pharmacological antibacterial tests were performed for both fractions.

The most active fraction (LTEtOAc-F, 31.9 g) was fractionated with a chromatographic open column (20 mm × 600 mm), previously packed with 140 g of silica gel 60 (Merck, mesh 70–230) as a stationary phase.

An *n*-hexane/EtOAc/MeOH gradient system was used as the mobile phase, with 10% polarity increments. Once the gradient system was 30:70 *n*-hexane:EtOAc, 100% of EtOAc was incorporated into the mobile phase; finally, 100% MeOH was added to the column.

Twenty-two samples (500 mL) were collected, which were grouped into seven final fractions (Ltc1-F1 to Ltc1-F7) according to their chemical composition. Ltc1-F3, Ltc1-F4, Ltc1-F5, and Ltc1-F6 were evaluated through a pharmacological antibacterial test.

The chemical separation was monitored by thin layer chromatography (TLC), and the plates were visualized under long (365 nm) and short (254 nm) UV lamps. All of them were developed with chromogenic developers.

The sub-fraction identified as Ltc1-F3 (5.3 g) was fractionated with a chromatographic open column (40 mm × 200 mm) and previously packed with silica gel 60 (Merck, mesh 70–230, 50 g).

An n-hexane/EtOAC/MeOH gradient system was used, increasing polarity with changes of 10%, obtaining 47 samples (50 mL) which were grouped into 20 fractions (Ltc2-F1 to Ltc2-F20).

The fraction identified as Ltc2-F9 (1.1059 g) showed a single spot in TLC; therefore, it was fractionated by chromatographic open column (reverse-phase) (40 mm × 200 mm, 10 g), packed with 10 g of reverse phase silica gel (LC-18 packing, SUPELCO), and as a mobile phase it was used with a water/acetonitrile gradient system with 5% polarity increments. In total, 51 samples (10 mL) were collected, which were grouped into 24 by similarity in contained compounds (Ltc3-F1-F24). Chemical analyses, through HPLC techniques, were conducted for LTHE, fractions, and sub-fraction Ltc1-F3.

The HPLC system consisted of a Waters 2695 separation module equipped with a Waters 996 photodiode array detector and Empower Pro Software (Waters Corporation, USA). A Supelcosil LC-F column (4.6 mm × 250 mm i.d., 5 µm particle size) (Sigma, Aldrich, Bellefonte, PA, USA) was used.

The mobile phase consisted of an aqueous solution of 0.5% trifluoracetic acid (solvent A) and acetonitrile (solvent B). The gradient system used was as follows: 0–1 min, 0%, B; 2–3 min, 5% B; 4–30 min, 30% B; 21–23 min, 50% B, 24–25% min 80% B; 26–27 min 100% B; 28–30 min 0% B. The flow rate was kept at 0.9 mL/min and the injection volume was 10 µL. The absorbance was measured at 280 nm and 350 nm.

The structures of compounds contained in fractions Ltc3-F9 and Ltc3-F15 were identified by analyses of the ^1^H and ^13^C nuclear magnetic resonance (NMR) spectra. The antibacterial activity of two pure compounds was determined.

### 3.5. Bacterial Strains and Culture Conditions

Antibacterial evaluations were performed against Gram-positive and Gram-negative bacteria, using reference strains (ATCC) of *Staphylococcus aureus*^6538^, *Listeria monocytogenes*^19113^, *Escherichia coli*^35218^, and *Pseudomonas aeruginosa*^9027^, as well as multidrug-resistant clinical isolates of *S. aureus*^01^, *S. aureus*^02^, *Bacillus cereus*, *E. coli*^01^, *E. coli*^02^, *Klebsiella pneumoniae,* and *Pasteurella multocida* from the Bacteriology Laboratory of the Academic Area of Veterinary Medicine and Zootechnics of the Autonomous University of Hidalgo State.

Bacterial strains were reactivated from cryopreservation in Müller–Hinton agar (BD Bioxon, Heidelberg, Germany). Gram staining was performed to corroborate their morphology and purity. One colony of each strain was inoculated in nutritive broth (BD Bioxon, Heidelberg, Germany), and incubated under constant agitation (70 RPM) for 24 h at 37 °C.

### 3.6. Antimicrobial Sensitivity Test of Field Strains

Antimicrobial sensitivity was tested only for clinical isolates. It was determined using the disk diffusion method in Müller–Hinton agar (BD Bioxon, Heidelberg, Germany), according to the methodology described by Rangel-López et al. (2022) [91].

A total of 100 µL of a bacterial suspension previously adjusted to a 0.5 McFarland standard (Remel, R20421, Lenexa, KS, USA) of each bacterium was inoculated and distributed on Petri plates. Each plate was allowed to dry for 15 min, and once this period had elapsed, multidiscs (PT-35, Mexico City, Mexico) were placed on the plate and incubated at 37 °C for 24 h.

After incubation, growth inhibition halos were measured and compared with measures established by the Clinical and Laboratory Standards Institute (CLSI) [44].

The antimicrobial active ingredients used were amikacin (30 µg), ampicillin (10 µg), carbenicillin (100 µg), cephalothin (30 µg), cefotaxime (30 µg), ciprofloxacin (5 µg), clindamycin (30 µg), chloramphenicol (30 µg), dicloxacillin (1 µg), erythromycin (15 µg), gentamicin (10 µg), netilmicin (30 µg), nitrofurantoin (300 µg), norfloxacin (10 µg), penicillin (10 U), sulfamethoxazole/trimethoprim (25 µg), tetracycline (30 µg), and vancomycin (30 µg).

### 3.7. Antibacterial Activity

The antibacterial activity of *Larrea tridentata* hydroalcoholic extract, fractions, sub-fractions, and compounds was determined through the minimal inhibitory concentration (MIC) and minimal bactericidal concentration (MBC) techniques, in accordance with the CLSI guidelines, and as described by Zaragoza-Bastida et al. (2020) [44,92].

#### 3.7.1. Minimal Inhibitory Concentration

The microdilution technique was used to determine MIC, and different concentrations were evaluated (100–0.78 mg/mL for LTHE; 50–0.19 mg/mL for LTAq-F and LTEtOAc-F, and 1.56–0.01 mg/mL for sub-fractions and compounds). Every concentration was prepared with nutritive broth, except for the organic fraction, sub-fractions, and compounds, which were solubilized in 15% and 10% dimethyl sulfoxide solvent (DMSO).

Into a sterile 96-well plate, 100 µL of each extract concentration was added, along with 10 µL of bacterial cell suspension previously adjusted to a 0.5 McFarland standard (Remel, R20421, Lenexa, KS, USA). Plates were incubated at 37 °C for 24 h at 70 rpm. Kanamycin (AppliChem 4K10421, Darmstadt, Germany) at different concentrations (128 to 0.125 µg/mL) and nutritive broth were used as positive and negative controls, respectively. Treatments were evaluated in triplicates.

After incubation, 20 µL of a 0.04% (*w*/*v*) p-iodonitrotetrazolium (Sigma-Aldrich I8377, St. Louis, MO, USA) solution was added into each well, and the plates were incubated for 30 min. The MIC was determined by the concentration at which the solution turned to a pinkish color.

#### 3.7.2. Minimal Bactericidal Concentration

After incubation and prior to addition of p-iodonitrotetrazolium, 5 μL from each well was inoculated in Müller–Hinton agar (DIBICO^®^ Mexico City, Mexico) and incubated at 37 °C for 24 h. The MBC was considered as the concentration at which no visible growth of the bacteria was observed on the plates.

To determine whether treatments had bactericidal or bacteriostatic effects, the ratio of MBC/MIC was determined. A bacteriostatic effect was considered when the ratio was greater than 4, and a bactericidal effect when values less than or equal to 4 were obtained [93].

### 3.8. Statistical Analysis

The obtained MIC and MBC data were normalized and analyzed using analysis of variance (ANOVA) to determine significant statistical differences between treatments. The differences between means were compared with Tukey’s test (*p* < 0.05) using SAS version 9.0 (SAS Institute, Cary, NC, USA).

## 4. Conclusions

The antibacterial activity of *L. tridentata* has been reported, and various compounds have been attributed to this activity. In the present investigation, the antibacterial activity of a hydroalcoholic extract of *L. tridentata*, two fractions, four sub-fractions, and two pure compounds were evaluated. The results indicated the pure compound *nor* 3′-demethoxyisoguaiacin, isolated from the organic fraction, could be responsible for the antibacterial activity of *L. tridentata*. *nor* 3′-demethoxyisoguaiacin exerts bactericidal activity against multidrug-resistant bacteria associated with bovine mastitis. Therefore, this compound could be used as an alternative treatment for this pathology, although in vivo studies remain necessary.

## Figures and Tables

**Table 1:** Results of inhibition halos (mm) and antimicrobial sensitivity of multidrug-resistant clinical isolates.

Antimicrobial	*S. aureus* ^01^	*S. aureus* ^02^	*B. cereus*	*E. coli* ^01^	*E. coli* ^02^	*K. pneumoniae*	*P. multocida*
**Amikacin**	15 (I)	**7 (R)**	20 (S)	**12 (R)**	**7 (R)**	**12 (R)**	21 (S)
**Ampicillin**	**7 (R)**	**7 (R)**	**7 (R)**	**11 (R)**	**7 (R)**	**7 (R)**	**7 (R)**
**Carbenicillin**	**22 (R)**	**15(R)**	**7 (R)**	**11 (R)**	**7 (R)**	**7 (R)**	25 (S)
**Cephalothin**	**26 (R)**	35 (S)	**11 (R)**	**12 (R)**	**7 (R)**	**7 (R)**	**7 (R)**
**Cefotaxime**	**14 (R)**	16 (I)	**8 (R)**	25 (I)	26 (S)	25 (I)	26 (S)
**Ciprofloxacin**	23 (S)	**11(R)**	20 (I)	22 (S)	25 (S)	30 (S)	29 (S)
**Clindamycin**	30 (S)	23 (S)	30 (S)	**14 (R)**	**7 (R)**	**7 (R)**	**7 (R)**
**Chloramphenicol**	25 (S)	20 (R)	20 (S)	**7 (R)**	**7 (R)**	21 (S)	27 (S)
**Dicloxacillin**	**7 (R)**	**7 (R)**	**7 (R)**	**7 (R)**	**7 (R)**	**7 (R)**	**7 (R)**
**Erythromycin**	16 (I)	24 (S)	25 (S)	**7 (R)**	**7 (R)**	**7 (R)**	**18 (R)**
**Gentamicin**	15 (S)	**12 (R)**	22 (S)	18 (S)	18 (S)	20 (S)	15 (S)
**Netilmicin**	**11 (R)**	**7 (R)**	15 (S)	**10 (R)**	17(S)	**10 (R)**	**12 (R)**
**Nitrofurantoin**	22 (S)	**7 (R)**	16 (I)	15 (I)	**7 (R)**	25 (S)	28 (S)
**Norfloxacin**	16 (I)	**7 (R)**	18 (S)	20 (S)	**7 (R)**	15 (I)	20 (S)
**Penicillin**	30 (S)	**7 (R)**	**7 (R)**	**7 (R)**	**7 (R)**	**7 (R)**	**7 (R)**
**Sulfamethoxazole/** **Trimethoprim**	**7 (R)**	**7 (R)**	18 (S)	**7 (R)**	27 (S)	16 (S)	20 (S)
**Tetracycline**	22 (S)	16 (I)	17 (I)	**7 (R)**	15 (S)	**7 (R)**	20 (S)
**Vancomycin**	**11 (R)**	**7 (R)**	17 (S)	**7 (R)**	**7 (R)**	**7 (R)**	**7 (R)**

I, intermediate; R, resistant; S, sensitive, in accordance with CLSI guidelines [44].

**Table 2:** Minimal inhibitory concentrations of *L. tridentata* against evaluated bacteria (mg/mL).

	Reference Strains				MDR Clinical Isolates						
Evaluated Treatment	01	02	03	04	05	06	07	08	09	10	11
**LTHE**	0.39 ^B,c^	3.12 ^D,d^	12.50 ^F,c^	12.50 ^F,c^	0.39 ^B,b^	0.19 ^A,e^	0.78 ^C,g^	6.25 ^E,c^	25.00 ^G,e^	6.25 ^E,c^	0.78 ^C,d^
**LTAq-F**	1.56 ^A,d^	1.56 ^A,c^	3.12 ^B,b^	3.12 ^B,b^	SA	SA	SA	12.5 ^D,d^	25.00 ^E,e^	6.25 ^C,c^	SA
**LTEtOAc-F**	0.04 ^A,a^	0.04 ^A,a^	3.12 ^D,b^	3.12 ^D,b^	0.39 ^C,b^	0.19 ^B,e^	0.19 ^B,e^	3.12 ^D,b^	3.12 ^D,c^	3.12 ^D,b^	0.39 ^C,c^
**Ltc1-F3**	0.09 ^B,b^	0.09 ^B,b^	1.56 ^E,a^	3.12 ^F,b^	0.02 ^A,a^	0.02 ^A,b^	0.02 ^A,b^	0.78 ^D,a^	0.78 ^D,b^	0.39 ^C,a^	0.09 ^B,a^
**Ltc1-F4**	0.09 ^C,b^	0.09 ^C,b^	3.12 ^G,b^	3.12 ^G,b^	0.02 ^A,a^	0.04 ^B,c^	0.04 ^B,c^	0.78 ^F,a^	0.78 ^F,b^	0.39 ^E,a^	0.19 ^D,b^
**Ltc1-F5**	0.04 ^B,a^	0.09 ^B,b^	3.12 ^E,b^	3.12 ^E,b^	0.02 ^A,a^	0.09 ^B,d^	0.09 ^B,d^	0.78 ^D,a^	0.78 ^D,b^	0.39 ^C,a^	0.09 ^B,a^
**Ltc1-F6**	0.09 ^A,b^	0.09 ^A,b^	3.12 ^D,b^	3.12 ^D,b^	0.78 ^C,c^	0.39 ^B,f^	0.39 ^B,f^	3.12 ^D,b^	6.25 ^E,d^	3.12 ^D,b^	0.78 ^C,d^
**C1**	NA	0.09 ^C,b^	1.56 ^F,a^	3.12 ^G,b^	0.02 ^B,a^	0.01 ^A,a^	0.01 ^A,a^	0.78 ^E,a^	0.39 ^D,a^	0.39 ^D,a^	0.09 ^C,a^
**C2**	NA	NA	NA	1.56 ^A,a^	NA	NA	NA	NA	NA	NA	NA
Kanamycin *	2	2	2	64	4	0.5	0.25	2	4	4	0.5
***p*-value**	0.0001										

MDR, multidrug-resistant; 01 *S. aureus*^6538^, 02 *L. monocytogenes*^19113^, 03 *E. coli*^35218^, 04 *P. aeruginosa*^9027^, 05 *S. aureus*^01^, 06 *S. aureus*^02^, 07 *B. cereus*, 08 *E. coli*^01^, 09 *E. coli*^02^, 10 *K. pneumoniae*, 11 *P. multocida*. NA, no activity; LTHE, *L. tridentata* hydroalcoholic extract; LTAq-F, aqueous fraction; LTEtOAc-F, organic fraction; Ltc1-F3, F4, F5, F6, sub-fractions from organic fraction; C1, compound 1 *nor* 3′-demethoxyisioguaiacin; C2, NDGA mixture; * Values are expressed in µg/mL. ^a,b,c,d,e,f^ Different letters in columns indicate significant statistical differences (*p* ≤ 0.05) between treatments, ^A,B,C,D,E,F,G^ Different letters in rows indicate significant statistical differences (*p* ≤ 0.05) between bacteria.

**Table 3:** ^1^H and ^13^C NMR data of *nor* 3′-demethoxyisoguaiacin (**1**, 600 and 150 MHz, CD_3_OD).

Position	δ^1^H (δ in ppm, J in Hz) 1	δ^13^C 1	Reported δ^13^C 1 ^a^
**1**		128.5	128.1
**2**	6.45 (s)	116.0	115.9
**3**		144.1	144
**4**		144.4	144.4
**5**	6.16 (s)	118.0	117.7
**6**		130.8	130.7
**7 a** **b**	2.75 (dd, 6.6, 16.1) 2.32 (dd, 5.1, 16.1)	35.9	35.7
**8**	1.92 (m)	30.5	30.1
**9**	0.82 (d, 5.1)	16.1	16.1
**1′**		139.7	139.3
**2′**	6.76 (d, 8.4)	130.9	130.8
**3′**	6.67 (d, 8.4)	115.6	115.7
**4′**		156.1	156.3
**5′**	6.67 (d, 8.4)	115.6	115.7
**6′**	6.76 (d, 8.4)	130.9	130.8
**7′**	3.46 (d, 6.2)	51.1	50.8
**8′**	1.81 (dd, 2.5, 6.6)	42.2	41.8
**9′**	0.8 (d, 6.2)	16.1	16.3

^a^ reported data by Bashyal et al. (2017) [28].

**Table 4:** Minimal bactericidal concentrations of *L. tridentata* against evaluated bacteria (mg/mL).

	Reference Strains				MDR Clinical Isolates						
Evaluated Treatment	01	02	03	04	05	06	07	08	09	10	11
**LTHE**	0.78 ^B,c^	6.25 ^D,c^	25.00 ^F,d^	25.00 ^F,c^	1.56 ^C,d^	0.39 ^A,d^	1.56 ^C,e^	25.00 ^F,d^	50.00 ^G,d^	12.50 ^E,d^	1.56 ^C,d^
**LTAq-F**	3.12 ^A,d^	3.12 ^A,b^	NA	NA	NA	NA	NA	NA	NA	NA	NA
**LTEtOAc-F**	0.09 ^A,a^	0.09 ^A,a^	6.25 ^D,c^	6.25 ^D,b^	0.78 ^C,c^	0.39 ^B,d^	0.39 ^B,c^	6.25 ^D,c^	6.25 ^D,c^	6.25 ^D,c^	0.78 ^C,c^
**Ltc1-F3**	NA	0.09 ^B,a^	NA	6.25 ^D,b^	0.09 ^B,b^	0.09 ^B,b^	0.04 ^A,a^	3.12 ^C,b^	3.12 ^C,b^	3.12 ^C,b^	0.19 ^C,a^
**Ltc1-F4**	NA	NA	6.25 ^E,c^	3.12 ^D,a^	0.09 ^B,b^	0.09 ^B,b^	0.04 ^A,a^	3.12 ^D,b^	3.12 ^D,b^	3.12 ^D,b^	0.39 ^C,b^
**Ltc1-F5**	0.19 ^B,b^	NA	3.12 ^D,b^	3.12 ^D,a^	0.09 ^A,b^	0.19 ^B,c^	0.09 ^A,b^	6.25 ^E,c^	6.25 ^E,c^	6.25 ^E,c^	0.78 ^C,c^
**Ltc1-F6**	0.09 ^A,a^	0.09 ^A,a^	6.25 ^E,c^	3.12 ^D,a^	1.56 ^C,d^	SA	0.78 ^B,d^	6.25 ^E,c^	6.25 ^E,c^	6.25 ^E,c^	1.56 ^C,d^
**C1**	NA	0.09 ^C,a^	1.56 ^F,a^	3.12 ^G,a^	0.04 ^B,a^	0.02 ^A,a^	0.78 ^E,d^	1.56 ^F,a^	1.56 ^F,a^	1.56 ^F,a^	0.19 ^D,a^
**C2**	NA	NA	NA	3.12 ^A,a^	NA	NA	NA	NA	NA	NA	NA
Kanamycin *	4	4	0.5	128	4	8	0.5	4	8	8	1
***p*-value**	0.0001										

MDR, multidrug-resistant; 01 *S. aureus*^6538^, 02 *L. monocytogenes*^19113^, 03 *E. coli*^35218^, 04 *P. aeruginosa*^9027^, 05 *S. aureus*^01^, 06 *S. aureus*^02^, 07 *B. cereus*, 08 *E. coli*^01^, 09 *E. coli*^02^, 10 *K. pneumoniae*, 11 *P. multocida*. NA, no activity; LTHE, *L. tridentata* hydroalcoholic extract; LTAq-F, aqueous fraction; LTEtOAc-F, organic fraction; Ltc1-F3, F4, F5, F6, sub-fractions from organic fraction; C1, compound 1 *nor* 3′-demethoxyisioguaiacin; C2, NDGA mixture; * Values are expressed in µg/mL. ^a,b,c,d,e^ Different letters in columns indicate significant statistical differences (*p* ≤ 0.05) between treatments, ^A,B,C,D,E,F,G^ different letters in rows indicate significant statistical differences (*p* ≤ 0.05) between bacteria.

## Data Availability

Data are contained within the article.

## Sample Availability

Samples of the compounds are not available from the authors.
